# Engineered mosaic protein polymers; a simple route to multifunctional biomaterials

**DOI:** 10.1186/s13036-019-0183-2

**Published:** 2019-06-18

**Authors:** Daniel T. Peters, Helen Waller, Mark A. Birch, Jeremy H. Lakey

**Affiliations:** 1 Institute for Cell and Molecular Biosciences, Medical School, Newcastle University, Newcastle upon Tyne, UK; 2 Division of Trauma and Orthopaedic Surgery, Department of Surgery, University of Cambridge, Cambridge, UK

**Keywords:** Biomaterials, Protein engineering, Tissue scaffolds, Synthetic biology, Bone, Electron microscopy

## Abstract

**Background:**

Engineered living materials (ELMs) are an exciting new frontier, where living organisms create highly functional materials. In particular, protein ELMs have the advantage that their properties can be manipulated via simple molecular biology. Caf1 is a protein ELM that is especially attractive as a biomaterial on account of its unique combination of properties: bacterial cells export it as a massive, modular, non-covalent polymer which is resistant to thermal and chemical degradation and free from animal material. Moreover, it is biologically inert, allowing the bioactivity of each 15 kDa monomeric Caf1 subunit to be specifically engineered by mutagenesis and co-expressed in the same *Escherichia coli* cell to produce a mixture of bioactive Caf1 subunits.

**Results:**

Here, we show by gel electrophoresis and transmission electron microscopy that the bacterial cells combine these subunits into true mosaic heteropolymers. By combining two separate bioactive motifs in a single mosaic polymer we demonstrate its utility by stimulating the early stages of bone formation by primary human bone marrow stromal cells. Finally, using a synthetic biology approach, we engineer a mosaic of three components, demonstrating that Caf1 complexity depends solely upon the variety of monomers available.

**Conclusions:**

These results demonstrate the utility of engineered Caf1 mosaic polymers as a simple route towards the production of multifunctional biomaterials that will be useful in biomedical applications such as 3D tissue culture and wound healing. Additionally, in situ Caf1 producing cells could create complex bacterial communities for biotechnology.

**Graphical abstract:**

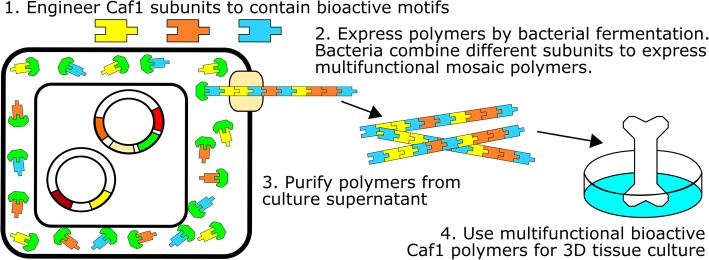

**Electronic supplementary material:**

The online version of this article (10.1186/s13036-019-0183-2) contains supplementary material, which is available to authorized users.

## Background

Engineered living materials (ELMs) are an exciting emergent field of study, where living organisms are critical components in the formation, maintenance or modulation of a material [1, 2]. In these systems, the organism is responsible for the processing of simple, sustainable raw ingredients into highly complex, functionalised “smart” materials, providing advantages over other systems which are either more time intensive, involve more expensive or less “green” reagents, or are less complex.

Proteins are of particular interest for development as ELMs [2]: evolution has caused the generation of proteins with a wide variety of finely tuned bioactivities and material properties ready to be exploited in different applications; and molecular biology techniques allow changes to be made at the sequence level through which these properties can be modified, or new properties introduced. For the most part, protein materials are based on those naturally available, such as collagen and spider silk that have favourable stiffness and bioactivity, and are in some cases engineered to further tailor their properties to particular applications [3–5]. However, recent studies have also began to focus on proteins naturally produced by bacteria, such as the Curli and Caf1 proteins from *Escherichia coli* and *Yersinia pestis* respectively. Curli is a component of *E. coli* biofilms, and forms an amyloid structure from monomeric CsgA protein subunits [1]. CsgA can be modified by the incorporation of peptide sequences that provide it with novel functions, such as silver nanoparticle templating and adhesion to stainless steel [6], as well as the SpyTag peptide [7], which allows the conjugation of larger proteins to the Curli fibres [6, 8]. Moreover, two component fibres containing alternating blocks of CsgA and His-tagged CsgA monomers could be produced and the patterning altered using chemical inducers [9]. These developments have led to exciting applications of Curli in nanotechnology [1, 6, 9], but the amyloid nature of these proteins has limited their potential use with mammalian cells and tissues.

Unlike Curli, Caf1 does not form amyloid structures and instead possesses an immunoglobulin-like fold [10]. Caf1 is a small ~ 15 kDa protein that is assembled by *Y. pestis* cells into long polymers that are megadaltons in size and can reach up to 1.5 μm in length [11]. The subunits are held together through strong, non-covalent interactions [10, 12] resulting in remarkable thermal and chemical stability [13]. Additionally, since Caf1 is exported from recombinant bacterial cells it can be economically produced, free from animal material. Finally, in its unmodified form it interacts weakly with mammalian cells [14, 15], possibly because Caf1 has evolved to shield *Y. pestis* from phagocytosis. These features combine to provide the key benefits of Caf1 – selected peptide sequences can be engineered into the protein’s inert structure to impart new properties, giving the robust, manufacturable polymers a precisely definable bioactivity [15]. Additionally, when reacted with a range of polyethylene glycol (PEG) cross-linkers Caf1 polymers can form hydrogels of tuneable stiffness and porosity [16], allowing the polymers to form a 3D scaffold. Therefore, Caf1 is particularly attractive for use as a biomaterial in biomedical applications such as 3D tissue culture and wound healing.

**Fig. 1:**
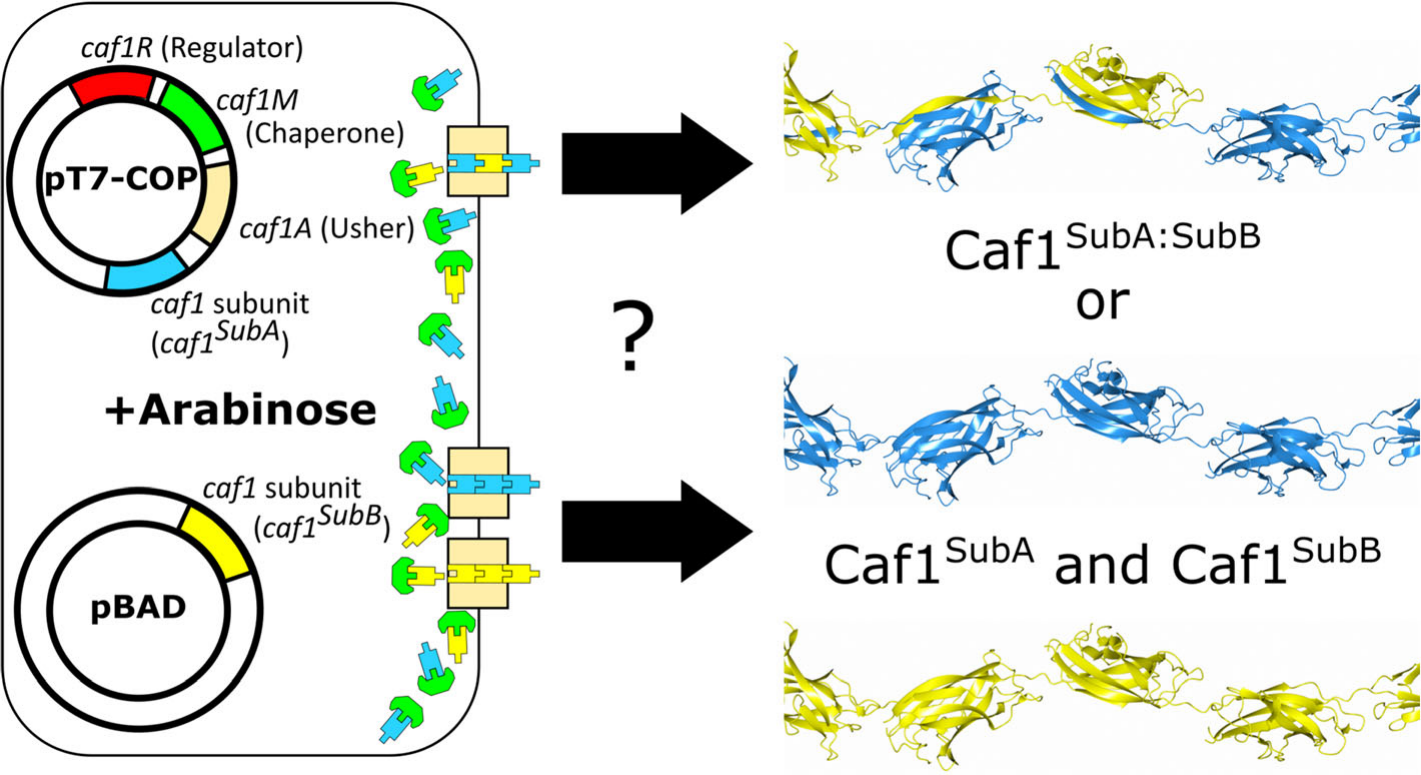
Production of mosaic Caf1 polymers by *E. coli*. In this diagram, *E. coli* have been transformed with the pT7-COP plasmid, which contains the *caf1* operon with a *caf1* subunit “A” gene (*caf1*^SubA^, blue), and a pBad plasmid containing a *caf1* subunit “B” gene (*caf1*^SubB^, yellow) under the control of an arabinose inducible promoter. When arabinose is added to cells growing in culture at 35 °C, both the *caf1* operon and the additional subunit genes are expressed. Subunits are exported to the periplasm where they are bound by the chaperone, Caf1M (green). Caf1M delivers the subunits to the outer membrane usher, Caf1A (tan), which assembles the subunits into a polymer. Both subunits have been detected in the extracellular fraction of cell cultures expressing both genes, but it was not known whether they form a mosaic homopolymer containing a mixture of the two subunits (Caf1^SubA^:^SubB^, top), or two separate homopolymers (Caf1^SubA^ and Caf1^SubB^, bottom). Caf1 polymer models were prepared from the Caf1:Caf1:Caf1M crystal structure (PDB: 1P5U), and visualised using the CCP4MG software package [50]

Caf1 polymers are members of the chaperone-usher family of proteins found in many Gram-negative bacteria. The monomeric subunits are synthesised in the cytoplasm and exported into the periplasm, via the Sec pathway [17], where they bind to a dedicated chaperone. The chaperone then transfers the subunits to the periplasmic end of the polymer by inserting their N-terminal β-strand into a vacant groove in the terminal subunit. As the polymers grow they are exported across the bacterial outer membrane via the usher protein. We have shown previously that if two versions of Caf1 subunit are co-expressed in the same cell they can both be detected in the resulting secreted Caf1 polymers [15]. However, it is not known whether these are two separate homopolymers, or a mosaic Caf1 heteropolymer (Fig. 1). Clearly, the ability to produce multi-functional Caf1 polymers would be advantageous, allowing the incorporation of multiple bioactive signals. Furthermore, the ability to dilute strongly bioactive or poorly expressing subunits with inactive wild-type subunits adds a unique degree of flexibility to the polymer production system.

In this work, we aimed to demonstrate the ability to produce true mosaic heteropolymers and assess the functionality of Caf1 mosaic polymers using bone tissue engineering as a case study. Bone possesses the ability to regenerate after damage [18]. However, in some cases, bone repair is not effective and the fracture fails to heal, necessitating a medical intervention [18–20]. One such intervention is the use of a tissue engineering strategy, where cell scaffolds are implanted at the injury site and expedite bone repair [20]. Such scaffolds can also be functionalised to release drugs, for example when used to restore bone loss following surgery to resect tumours such as osteosarcoma [21]. In addition these materials have the potential to be exploited in the formation of 3D in vitro models of bone disease [22]. Two important signals in bone tissue engineering are osteopontin (OPN) and bone morphogenetic protein 2 (BMP2). OPN provides adhesion sites to cells through integrin attachment [23] and can stimulate angiogenesis both in vitro and in vivo [24–26], whereas BMP2 has an important role in the differentiation of cells into osteoblasts [27–29]. Previously, the incorporation of OPN and BMP2 peptide sequences into a protein scaffold facilitated the adhesion of primary rat osteoblasts, prevented their de-differentiation and supported mineral deposition in the absence of any extra factors [30]. Therefore, the combination of these two motifs as bioactive modules within a single Caf1 polymer represents an interesting exemplar for determining whether multifunctional mosaic Caf1 polymers can be produced.

Here, we demonstrate that the co-expression of Caf1 subunits leads to mosaic heteropolymers using five *caf1* mutants and two independent approaches; SDS-PAGE and electron microscopy. We then co-express Caf1 mutant subunits harbouring the OPN and BMP2 peptide sequences at their N-termini to create a mosaic OPN:BMP2 heteropolymer. Cell biology experiments then show that this mosaic polymer can induce the early stages of bone formation by primary human bone marrow stromal cells (hBMSCs). Finally, using a synthetic biology approach, we engineer an extra Caf1 gene into our expression plasmid and demonstrate the production of a 3-subunit mosaic Caf1 polymer, suggesting that the system could be expanded to create even more complex mosaic polymers. These results demonstrate the major advantages of the ELM system of Caf1 production – through the genetic modification of the bacterial cells responsible for Caf1 biogenesis, complexity and control over the final material can be introduced without substantial hands-on input from the user. This provides a simple route towards the creation of multifunctional biomaterials, where different bioactivities can be engineered and combined to produce highly functionalised materials in a designed manner for use in applications such as 3D tissue culture and wound healing. Additionally, Caf1 expressing cells may be useful for in situ creation of engineered biofilms for biotechnology, as has been shown previously with other proteinaceous ELMs such as Curli and S-layers [1, 2].

## Results

### Generation of reduced stability Caf1 polymers

To detect if mosaic heteropolymers are produced, it was necessary to generate a variant whose properties differentiated it from the wild-type subunit (Caf1^WT^). Caf1 has a high thermostability [13], and a previous study has shown that mutagenesis of single amino acids in the N-terminal strand reduces the stability of Caf1 oligomers in the periplasm of *E. coli* [12]. In particular, the A5I substitution did not appear to inhibit formation of Caf1 oligomers, but reduced the apparent melting temperature from ~ 75–85 °C to ~ 65 °C. Subunits with a lower stability offered a way to determine whether co-expression of Caf1 subunits results in the production of Caf1 mosaic heteropolymers.

**Fig. 2:**
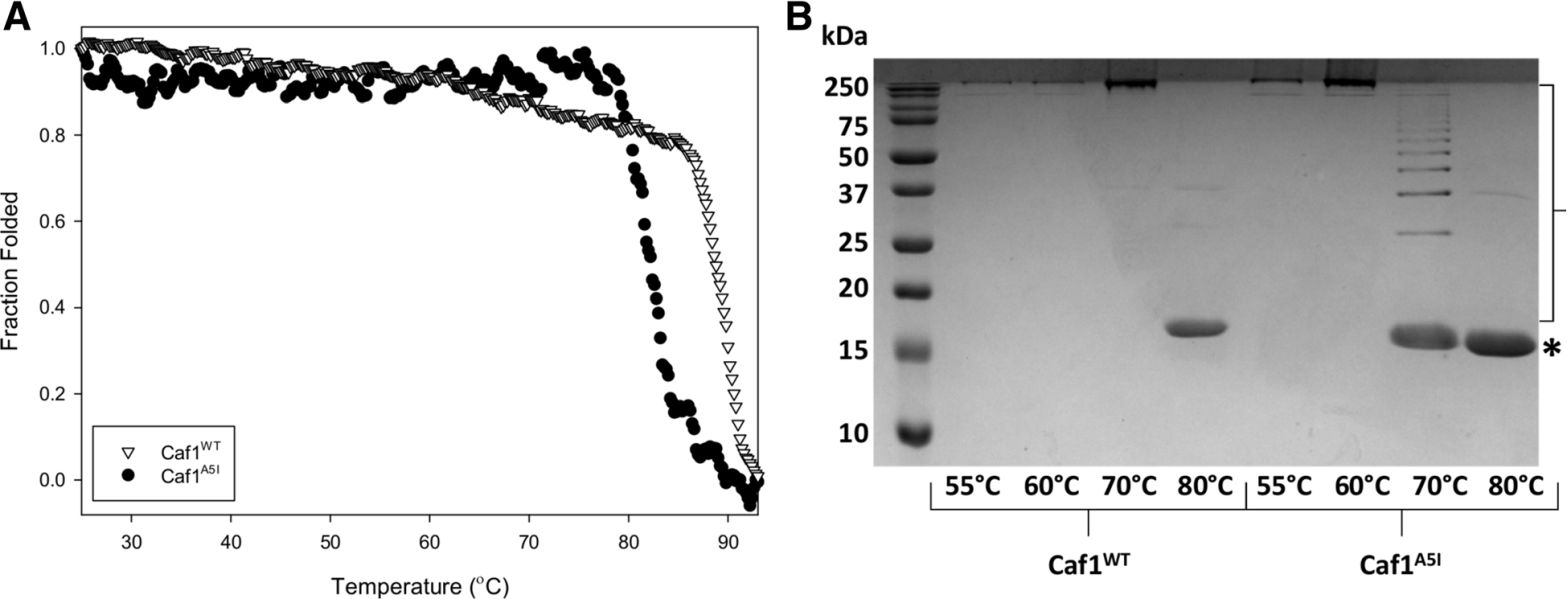
Characterisation of the Caf1^A5I^ low stability mutant. (**a**) Near-UV circular dichroism thermal melt of the Caf1^WT^ (adapted from [13]) and Caf1^A5I^ proteins. Circular dichroism at 290 nm was followed as 1 mg/mL protein was heated between 25 and 95 °C, and the signal converted into fraction of protein folded. Data represent the average of three independent replicates. (**b**) SDS-PAGE analysis of Caf1^WT^ and Caf1^A5I^ proteins heated for 5 min in SDS containing sample buffer at the indicated temperatures. The position of the monomeric species is denoted by a star, with oligomeric breakdown products seen as a ladder within the region bounded by the bracket

Therefore, *E. coli* BL21 cells were transformed with pT7-COPΔR-Caf1^A5I^ plasmid, which contains the Caf1 biosynthetic genes *caf1M* and *caf1A*, as well as the mutant *caf1* subunit *caf1*^*A5I*^, all under the control of a T7 promoter. The production of Caf1^A5I^ homopolymers (Additional file 1: Table S1) was confirmed by SDS-PAGE analysis (Additional file 1: Figure S1). To determine whether the melting temperature of the mutant polymers was indeed lower, the cooperative thermal transitions of the proteins were measured by circular dichroism (CD) (Fig. 2A). The “melting temperature” (mid-point of the thermal transition T_m_) was determined to be 82.2 ± 0.6 °C, 7.5 °C lower than the melting temperature of the wild-type protein (89.7 ± 0.4 °C [13]) measured under the same conditions. Next, the proteins were incubated in SDS-PAGE sample buffer at different temperatures for 5 min before analysis on SDS-PAGE. Unheated Caf1^A5I^ polymer does not enter the gel due to its large size but broke down into a visible ladder of oligomers at 70 °C, whereas the Caf1^WT^ polymer remained intact until 80 °C (Fig. 2B). Therefore, the Caf1^A5I^ mutant subunit forms long Caf1 polymers with a lower stability than the wild-type protein.

### Co-expression of *caf1* subunits leads to mosaic Caf1 polymers

To determine whether co-expression of *caf1* subunits leads to mosaic heteropolymers, we employed the use of another Caf1 mutant subunit containing a hexa-histidine tag joined by a flexible linker sequence to the N-terminus of Caf1 (Caf1^His^, Additional file 1: Table S1). This mutant has a molecular weight 1.6 kDa higher than that of Caf1^WT^, and so can be resolved from both the wild-type and Caf1^A5I^ subunits by SDS-PAGE. Importantly, Caf1^His^ has a wild-type N-terminal β-strand, and so will form the same subunit-subunit interface as the wild-type protein. Therefore, the Caf1^His^, like Caf1^WT^, is stable and will not break down into oligomers when heated to 70 °C for 5 min. Interestingly, Caf1^His^ homopolymers could not be purified in usable quantities, but could be co-expressed with either Caf^WT^ or Caf1^A5I^ subunits.

**Fig. 3:**
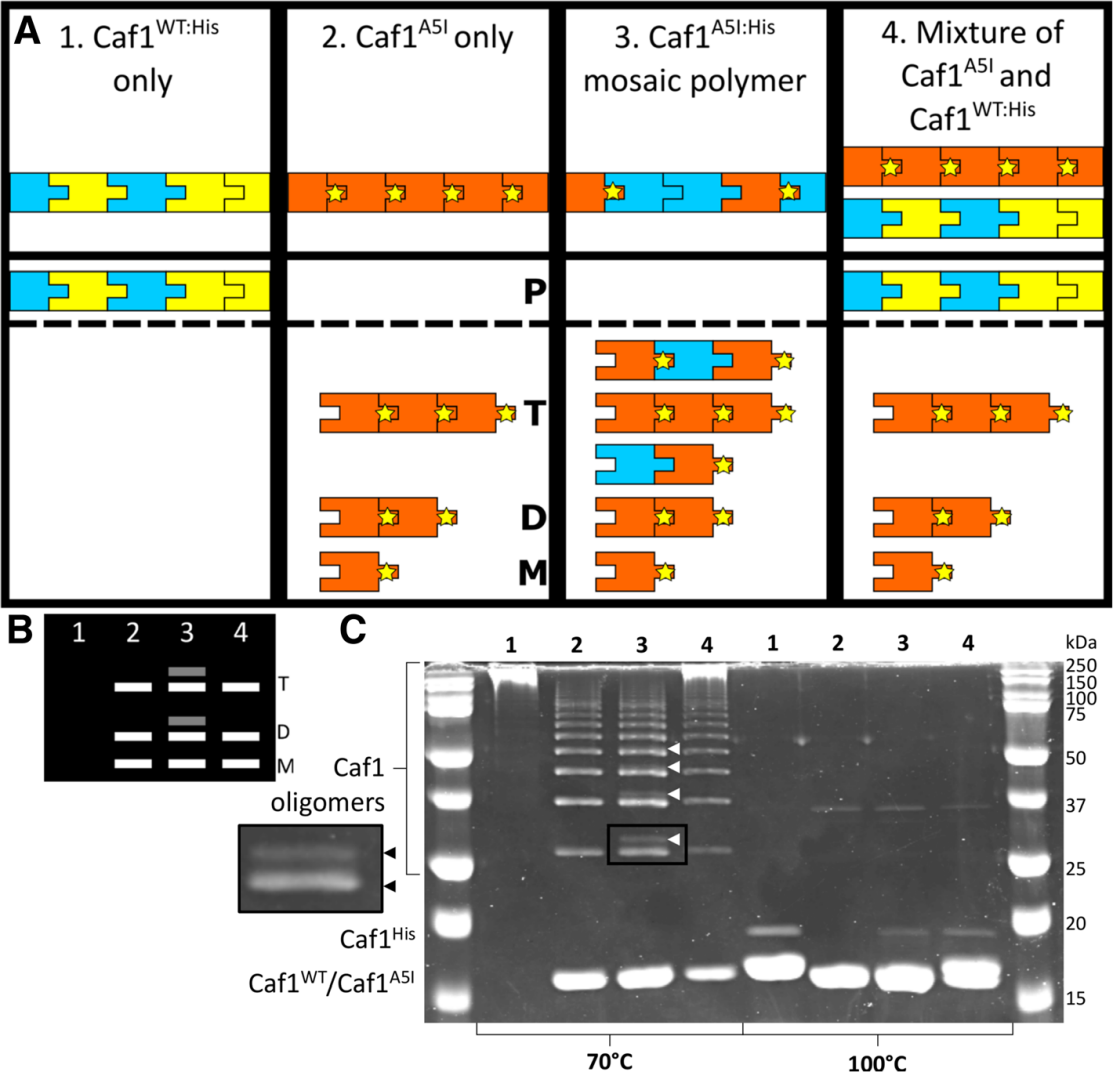
Characterisation of Caf1 mosaic polymers by SDS-PAGE. (**a**) Three mutant Caf1 subunits are depicted as cartoons: Caf1^WT^ in yellow, Caf1^His^ in blue and Caf1^A5I^ in orange, with a yellow star showing the location of the A5I mutation in the N-terminal β-strand that forms the subunit-subunit interface. Caf1^WT: His^, Caf1^A5I^ and Caf1^A5I: His^ proteins, as well as a mixture of Caf1^A5I^ and Caf1^WT: His^, are shown in the top panel, with their expected state when heated to 70 °C shown in the bottom panel. Species which span the box represent full length Caf1 polymers. At 70 °C, the Caf1^A5I^ subunit-subunit interactions break, leading to a pattern of oligomers (Monomer – M, Dimer – D, Trimer – T). The Caf1^WT^ and Caf1^His^ subunits have wild-type subunit-subunit interfaces, and do not break down at 70 °C (Polymer – P). If the Caf1^A5I^ subunit forms mosaic heteropolymers when co-expressed with the Caf1^His^ subunit, extra bands corresponding to oligomers containing the higher molecular weight Caf1^His^ subunit will be present. (**b**) Expected gel result if co-expression of Caf1^A5I: His^ subunits leads to mosaic heteropolymers. The expected monomer (M), dimer (D) and trimer (T) bands are shown as lines for samples 1–4 from (**a**) when they are heated at 70 °C. If the co-expressed subunits form separate homopolymers, samples 2, 3 and 4 would appear identical. (**c**) SDS-PAGE analysis of Caf1 mosaic polymers. Four samples consisting of a Caf1^WT:His^ mosaic polymer, a Caf1^A5I^ mutant Caf1 polymer, an Caf1^A5I:His^ mosaic polymer and an equimolar mixture of the Caf1^WT:His^ and Caf1^A5I^ polymers, were heated in SDS sample buffer for 5 min at either 70 °C or 100 °C. The Caf1^A5I^ and Caf1^WT^ subunits have similar molecular weights (15.6 kDa), whereas the Caf1^His^ subunit has a molecular weight which is ~ 2 kDa higher (17.2 kDa). The positions of these monomeric subunits are highlighted, and oligomeric breakdown products can be observed within the region bounded by the bracket. Oligomeric breakdown products containing the higher molecular weight Caf1^His^ subunits in the Caf1^A5I:His^ mosaic polymer sample are highlighted using small white triangles, with the dimer region from sample 3 expanded and shown to the left of the gel

Three *E. coli* expression cultures were set up and the following Caf1 polymers purified: (1) containing both Caf1^WT^ and Caf1^His^ subunits, (2) only the Caf1^A5I^ subunit and (3) both Caf1^A5I^ and Caf1^His^ subunits. Samples (1), (2) and (3) were then prepared for SDS-PAGE, as well as (4), which is an equimolar mixture of (1) and (2). The samples were then incubated in SDS-PAGE loading buffer at either 70 °C or 100 °C for 5 min and subsequently analysed by SDS-PAGE. After incubation at 100 °C, all polymers will denature into their constituent monomers, allowing the monomer composition to be ascertained. At 70 °C, the pattern of bands, corresponding to the breakdown of the polymers into oligomers, will depend upon whether the co-expression of the *caf1* subunits leads to separate homopolymers or to mosaic heteropolymers, according to the following rationale (Fig. 3A and B):

(i) At 70 °C, for sample (1), as both Caf1^WT^ and Caf1^His^ subunits have wild-type stability, they will remain polymeric and not enter the gel.
(ii) For sample (2), the A5I mutation destabilises the interface, and so should lead to the breakdown of the polymer into a ladder of oligomers, as in Fig. 2B.
(iii) For sample (3), if the subunits form separate polymers the result will be the same as (ii) since the Caf1^His^ will not break down, leaving only a ladder of Caf1^A5I^ monomers, dimers, trimers etc. If a heteropolymeric mosaic is present some of the oligomers will contain one or more heavier Caf1^His^ subunits, leading to additional bands on the SDS-PAGE. This will be most obvious at the dimer level where Caf1^A5I^:Caf1^His^ dimers will be clearly larger than Caf1^A5I^:Caf1^A5I^ dimers and Caf1^His^:Caf1^His^ dimers will not be formed.
(iv) The control, sample (4), mimics the situation expected if co-expressed subunits form separate homopolymers, and if this is the case then samples (2), (3) and (4) should appear identical on the gel (Fig. 3A).

The SDS-PAGE analysis (Fig. 3C) revealed that, at 100 °C, all polymers were completely denatured into their monomeric subunits, revealing either one monomer band for the Caf1^A5I^ homopolymer, or two bands for samples containing either Caf1^A5I^ or Caf1^WT^ with Caf1^His^. After treatment at 70 °C, in sample (1) the Caf1^WT^^:^^His^ protein remained polymeric and did not enter the gel due to its size, showing no breakdown into oligomers. In sample (2), the Caf1^A5I^ polymer broke down into the expected pattern of oligomeric species already seen in Fig. 2B. In sample (3), the oligomeric breakdown products were again observed, but in this case clear additional higher molecular weight bands could be observed for the dimer, trimer and tetramer bands before the gel resolution made any further bands difficult to detect. Sample (4) appeared identical to sample (2), apart from a bright band near the top of the gel corresponding to the Caf1^WT^ and Caf1^His^ subunits, which do not form oligomers at 70 °C. Therefore, the presence of the extra bands in sample (3) which are not present in samples (2) and (4) demonstrates that co-expression of Caf1 subunit leads to mosaic heteropolymers.

**Fig. 4:**
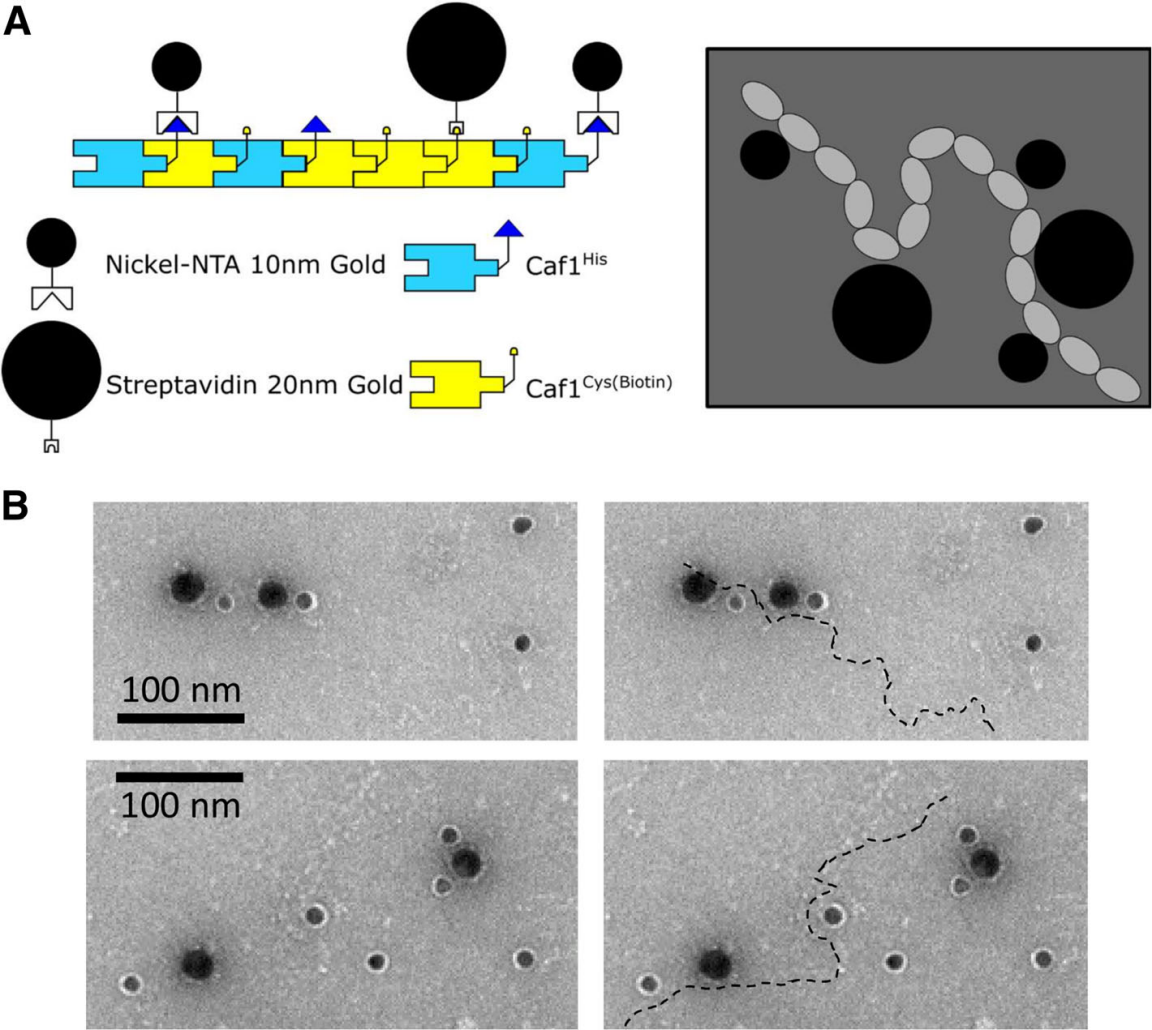
Characterisation of Caf1 mosaic polymers by transmission electron microscopy (**a**) Schematic of the electron microscopy experiment. Mosaic Caf1 polymers containing a His tag (blue, Caf1^His^) and a biotinylated cysteine (yellow, Caf1^Cys(Biotin)^) are mixed with Nickel-NTA-10 nm and Streptavidin-20 nm gold conjugates. These recognise and bind to the His tags (blue triangles) and biotinylated cysteines (yellow semicircles) respectively. On the electron micrograph (grey box), these should appear as light grey beads (Caf1 polymer) surrounded by small and large black dots, representing the two different size gold conjugates. (**b**) Negative stain electron micrographs of the His-tagged, biotinylated cysteine containing mosaic polymer (Caf1^His:Cys(Biotin)^). His-tagged subunits were labelled with Nickel-NTA-10 nm gold particles and biotinylated cysteine containing subunits labelled with Streptavidin-20 nm gold particles. Images were taken at 92000x magnification. Dotted lines show the position of the Caf1 polymer. Additional data for WT unlabelled polymers are shown in Supplementary Information

To test the presence of mosaic polymers by a second independent method, we co-expressed a mixture of two Caf1 subunits, one harbouring a single inserted cysteine residue not present in wild-type Caf1 (Caf1^Cys^, Additional file 1: Table S1) and the Caf1^His^ subunit and purified (Caf1^His:Cys^). The cysteine was then biotinylated with biotin-maleimide. The Caf1^His:Cys(Biotin)^ polymer was then adsorbed onto a nickel transmission electron microscopy (TEM) sample grid and probed using a combination of 10 nm (Nickel-NTA) and 20 nm (streptavidin) gold nanoparticle conjugates to selectively label Caf1^His^ and Caf1^Cys(Biotin)^ respectively. When visualised by negative stain TEM, the Caf1 polymers looked like beads on a string, as described previously [11]. Many 20 nm and 10 nm gold particles could be seen to associate with these polymers, with individual polymers binding both sizes of gold (Fig. 4, Additional file 1: Fig. S2). In contrast, when the Caf1^WT^ was prepared and analysed in the same way, very few gold particles were observed and these did not appear to associate with the Caf1 polymers (Additional file 1: Figure S2 C and D). This provides further evidence that co-expression of two different *caf1* genes in *E. coli* results in Caf1 mosaic heteropolymers, rather than separate homopolymers.

### Mosaic Osteopontin:BMP2 Caf1 polymers direct the early formation of bone by primary human stem cells

Having demonstrated that Caf1 mosaic polymers contain both types of subunit, we sought to determine whether this property could be used to add two separate bioactivities to a single Caf1 mosaic polymer. Previously, peptide motifs that mimic the action of osteopontin and bone morphogenetic protein 2 (OPN and BMP2 respectively) were shown to have differential effects on osteoblasts when incorporated into a protein scaffold, and together facilitated bone formation [30]. The OPN motif facilitated the adhesion, spreading and vinculin expression of primary rat osteoblasts, whilst BMP2 had little effect. On the other hand, incorporation of the BMP2 motif into the protein triggered SMAD (Sma and MAD related protein) signalling and matrix mineralisation, corresponding to the osteogenic differentiation of the cells, whereas OPN had little effect in this regard. Critically, when the cells were grown on a surface containing different proportions of OPN containing protein in the presence of recombinant BMP2 protein, the degree of SMAD signalling was dependent on the levels of cell adhesion, showing that these two motifs can be used synergistically. This system therefore represented an appealing test case for determining the utility of Caf1 mosaic polymers.

**Fig. 5:**
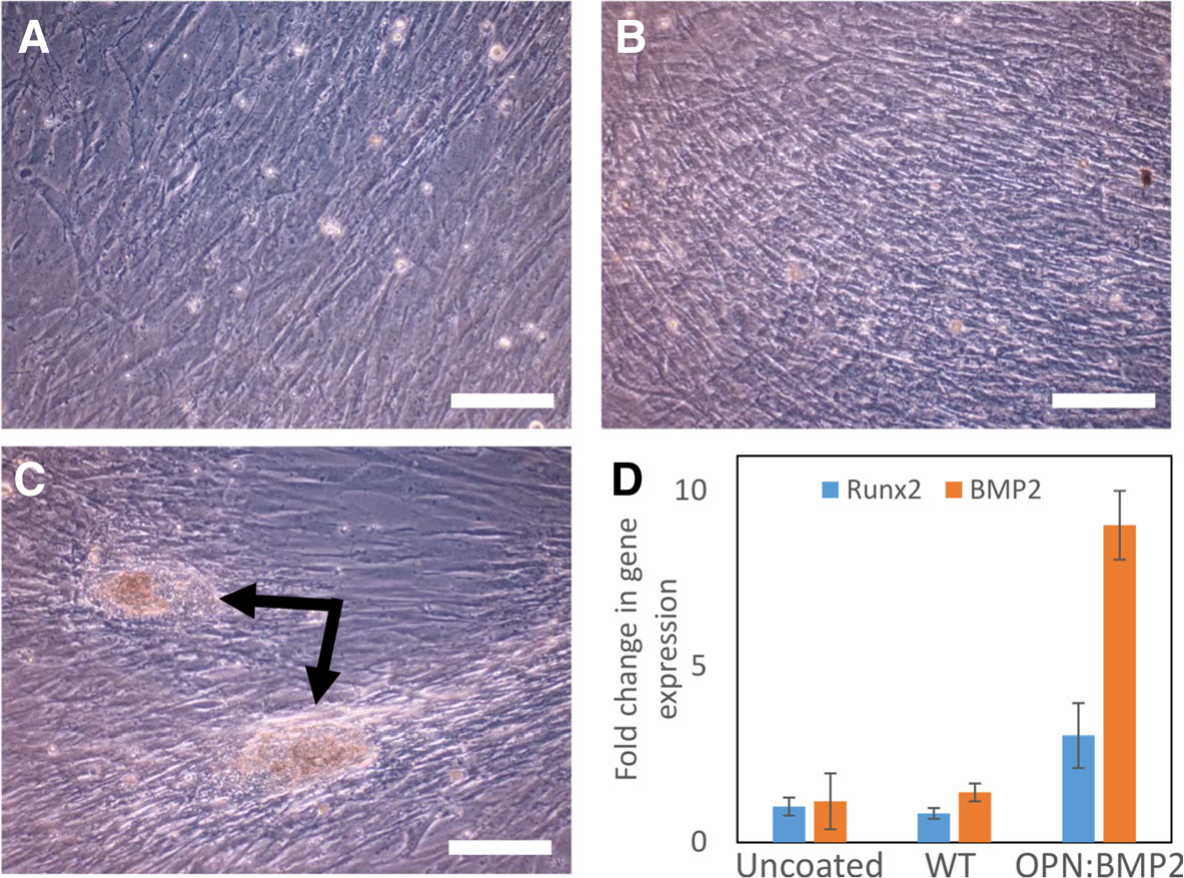
Caf1 mosaic polymers direct the early stages of bone formation. Phase microscopy images of primary human bone marrow stromal cells grown on plastic surfaces that were either uncoated (**a**) or coated with Caf1^WT^ (**b**) or Caf1^OPN:BMP2^ (**c**) mosaic polymers (scale bar = 200 μm). Areas of mineralisation triggered by the differentiation of the cells are highlighted with black arrows. (**d**) qRT-PCR analysis of Runx2 (blue) and BMP2 (orange) expression from each of the cultures at 14 days. Error bars represent the standard deviation from three biological replicates

Mutant Caf1 subunits harbouring the OPN and BMP2 sequences at their N-termini were generated (*caf1*^*OPN*^ and *caf1*^*BMP2*^, Additional file 1: Table S1). The corresponding plasmids, pT7-COP-Caf1^OPN^ (containing the *caf1* operon, where the *caf1* subunit is substituted by the *caf1*^*OPN*^ mutant), and pBad-Caf1^BMP2^ (where the *caf1*^*BMP2*^ subunit is under the control of an arabinose inducible promoter) were used to co-transform *E. coli* cells. A mosaic Caf1^OPN:BMP2^ polymer was then expressed by growing the cells at 35 °C in the presence of arabinose, and subsequently purified. Primary hBMSC’s were then grown in a 24-well plate, using either untreated, Caf1^WT^ or Caf1^OPN:BMP2^ surfaces as substrates. The cells were cultured for 14 days in the absence of any osteogenic supplements, and analysed by phase microscopy and qRT-PCR. The results showed that the mosaic Caf1 polymer triggered the osteogenic differentiation of the cells and the early stages of bone formation, as evidenced by distinct patches of mineralisation, which were not present on either the untreated of Caf1^WT^ surfaces (Fig. 5A-C). Moreover, qRT-PCR analysis revealed a large increase in the expression of Runx2 and BMP2, which are markers of osteogenic differentiation, in the presence of the mosaic polymer but not the Caf1^WT^ or untreated surfaces (Fig. 5D). These results demonstrate that the ability to trigger osteogenic differentiation of human primary cells has been specifically engineered into the Caf1 mosaic polymer.

### Engineering a 3-subunit Caf1 mosaic polymer

**Fig. 6:**
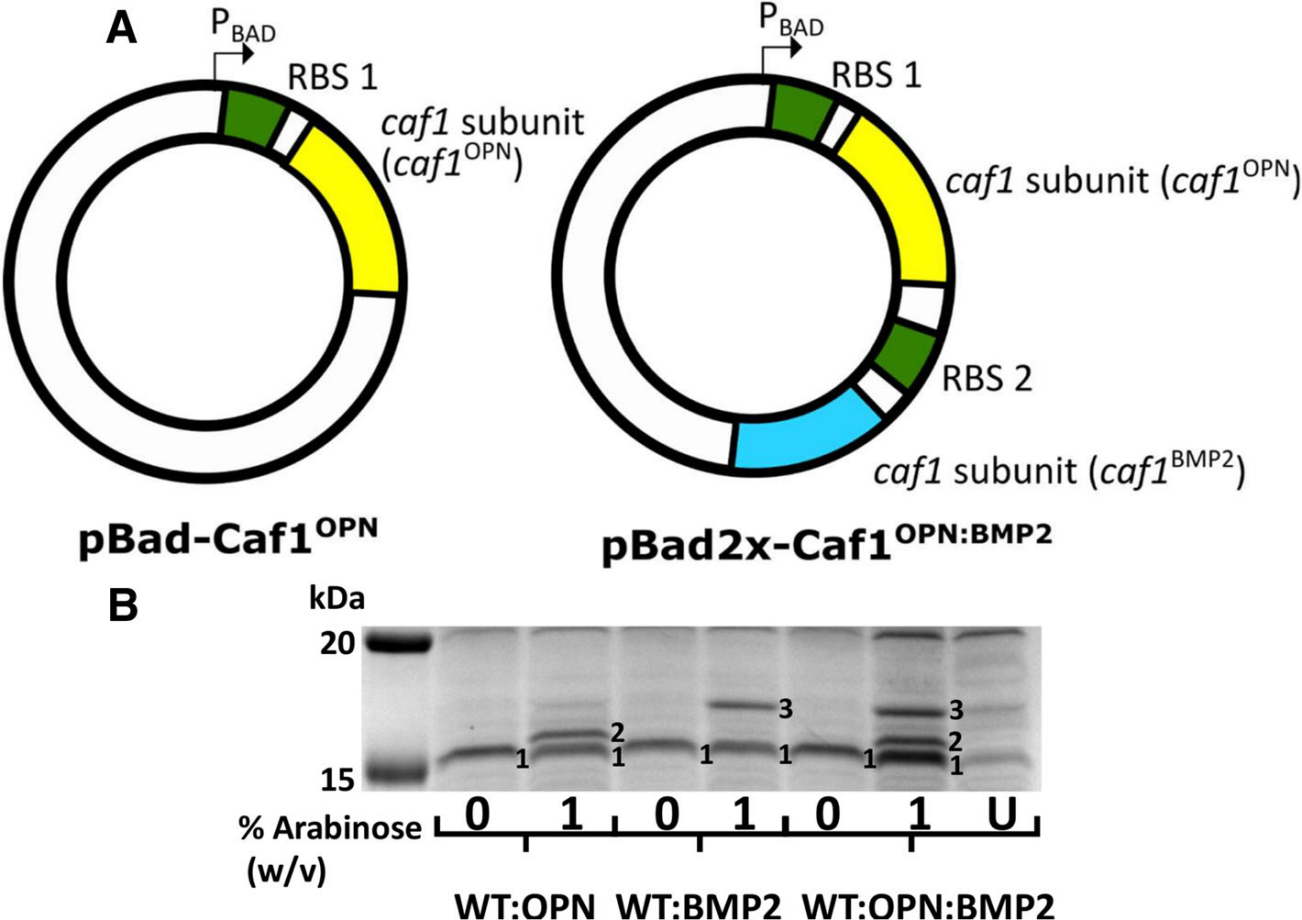
Design and production of a 3-subunit mosaic Caf1 polymer. (**a**) Diagram depicting the pBad-Caf1^OPN^ and pBad2x-Caf1^OPN:BMP2^ plasmids used in this study. The pBad plasmid was used as a template for the insertion of a *caf1* mutant gene containing the osteopontin sequence (Caf1^OPN^, yellow), a second ribosome binding site (RBS, green) and a *caf1* mutant gene containing the BMP2 peptide sequence (Caf1^BMP2^, blue) in order to construct the pBad2x-Caf1^OPN:BMP2^ plasmid, which was thus designed to express two *caf1* genes at once under the control of the arabinose inducible promoter. The pBad-Caf1^OPN^ plasmid, designed to express only the one *caf1* gene, is shown alongside as a comparison. (**b**) SDS-PAGE analysis showing the expression of a 3-subunit mosaic Caf1 polymer. Cultures of *E. coli* BL21(DE3) cells transformed with pCOP and either pBad-Caf1^OPN^, pBad-Caf1^BMP2^ or pBad2x ^OPN:BMP2^ were grown for 22 h in the presence and absence of 1% w/v arabinose. Samples of the extracellular fraction (flocculent layer and supernatant) were then heated to 100 °C for 5 min in SDS containing buffer and applied to the gel. The “U” lane represents the pCOP/pBad2x-Caf1^OPN:BMP2^ 1% arabinose sample that was not heated to 100 °C before application, and shows most Caf1 subunits are present in high molecular weight polymers. The monomeric subunits corresponding to each mutant are shown by numbers next to the relevant band: 1 is Caf1^WT^, 2 is Caf1^OPN^ and 3 is Caf1^BMP2^

To demonstrate the potential of Caf1 mosaic polymers, we sought to engineer a 3-subunit mosaic polymer using a plasmid design that can accommodate multiple subunit genes. To achieve this, we aimed to insert an extra Caf1 coding sequence with its own ribosome binding site into the pBad plasmid immediately following the first *caf1* gene so that both mutants could be translated from a single mRNA transcript driven by the same promoter. The size of the pBad plasmid, which lacks the chaperone and usher genes present in pT7-COP, makes genetic manipulation relatively straightforward. The first and second ribosome binding sites had the same sequence so that both subunits would be translated at the same rate. The Caf1^OPN^ and Caf1^BMP2^ mutant subunits were chosen as candidate proteins because of their sizes (16.8 kDa and 17.6 kDa respectively, compared to Caf1^WT^; 15.6 kDa). This would allow them to be distinguished when resolved by SDS-PAGE. The resulting plasmid, consisting of a pBad backbone, with an arabinose inducible transcriptional unit containing two independently translated Caf1 mutants was called pBad2x-Caf1^(OPN:BMP2)^ (Fig. 6A). *E. coli* cells were then co-transformed with pBad2x-Caf1^(OPN:BMP2)^ along with a plasmid containing either the full Caf1 operon (pCOP) or the operon with a T7 promoter upstream (pT7-COP). In this system, the main pCOP/pT7-COP plasmid supplies the genes of the Caf1 operon (*caf1R*, *caf1M*, *caf1A* and *caf1*^*WT*^) and the second supplies the two mutant Caf1 subunits. Cultures containing either 0% or 1% w/v arabinose were grown for 22 h at 35 °C and proteins resolved on SDS-PAGE. For the pT7-COP containing cultures, large amounts of Caf1^WT^ could be observed, however, in the presence of arabinose, bands corresponding to the mutant subunits could not be seen (Additional file 1: Figure S3). When pCOP, which expresses the *caf1* operon from its native promoters, was used as the main plasmid, the addition of arabinose to induce pBad2x-Caf1^(OPN:BMP2)^ resulted in the clear production of two additional bands of higher molecular weight than Caf1^WT^ (Fig. 6B). Comparison of these bands with those produced by cultures expressing 2-subunit mosaic polymers of Caf1^WT:OPN^ and Caf1^WT:BMP2^ allowed the bands in the 3-subunit system to be positively identified. Therefore, the use of the engineered pBad2x plasmid allows the production of a 3-subunit mosaic Caf1 polymer when co-transformed with the pCOP plasmid.

## Discussion

### N-terminal strand mutations result in lower stability Caf1 polymers

The basis for Caf1’s high thermal stability is the tight, non-covalent interaction between subunits, where small hydrophobic residues on the N-terminal β-strand of one subunit slot into pockets in the body of the next subunit [12]. Mutation to larger hydrophobic residues reduced the thermostability of Caf1 periplasmic oligomers [12]. Our objective was to create a Caf1 polymer with lower stability to allow us to differentiate it from the wild-type protein. We selected the A5I mutation as the most promising candidate from the previous study, as it caused a drop in Caf1’s unusual thermostability without affecting its ability to oligomerise. Here, we show that the A5I mutation permits the production of high molecular weight (> 500 kDa) Caf1 polymers, but with a lower thermal stability. This mutation was of particular utility in this study, but the production of Caf1 polymers of reduced stability could be of further use, for example in enhancing the biodegradability of implanted cell scaffolds.

### Co-expression of Caf1 subunits leads to mosaic heteropolymers

It had been observed that producing different Caf1 subunits on two separate plasmids results in the simultaneous production of the two types of Caf1 in polymeric form [15]. It was not known whether these were mosaic heteropolymers, containing a random mixture of both types of subunit, or two separate types of polymers due to a possible sorting mechanism within the cell. Here, we have demonstrated that these subunits are assembled together in a random mosaic heteropolymer, as evidenced by the differential breakdown products at 70 °C between the Caf1^A5I^ homopolymer, the Caf1^A5I:His^ mosaic polymer and a mixture of the two separate Caf1^A5I^ and Caf1^WT:His^ polymers, as well as the observation of Caf1^His:Cys^ mosaic polymers by transmission electron microscopy.

The yields of the mosaic polymers produced here were lower than those of the single subunit polymers (~ 22 mg/L vs. ~ 200 mg/L [13]). This could be due to the inclusion of the Caf1^His^ subunit, which could not be expressed on its own and may be more challenging for the cells to express and incorporate into polymers than the wild-type subunit. Further optimisation of the production process may help to improve these yields.

The dissociation constant (K_d_) of the Caf1 subunit:subunit interaction has been estimated to be at least 10^− 14^ M, which would place it amongst some of the tightest known interactions known [31]. Moreover, the interaction between FimG and FimF (homologous pilin subunits from *E. coli*) has a half-life of 3 × 10^9^ years, providing it with an “infinite stability against dissociation” [32]. Therefore, it is unlikely that the Caf1 subunits, once formed into a polymer, would be able to dissociate spontaneously, eliminating the possibility of subunit exchange within the polymer. Indeed, even the subunits on the ends of the polymer associate via the same donor strand complementation mechanism and so are likely to remain stably incorporated. Therefore, polymer growth can only take place through the addition of subunits to the growing ends of a polymer chain.

There are potentially significant advantages to using mosaic Caf1 polymers over conventional single subunit polymers, or even mixtures of single subunit polymers. Firstly, multiple functionalities, such as adhesion and differentiation, can be combined into a single material or hydrogel, as in our Caf1^OPN:BMP2^ mosaic polymer. Secondly, as the production of the pBad encoded subunit appeared to be at a lower level than the pT7-COP encoded subunit, bioactive motifs can be diluted by co-expressing them with the inert, non-stick Caf1^WT^ [15]. The density of adhesion motif can affect the migration and proliferation of cells, and previous studies have shown that intermediate densities tend to give optimal values for these processes [33–37]. Expressing Caf1 polymers as mosaics provides the ability to control the motif density. This may also be of use in the manufacture of Caf1 polymers since it is clear that some large modifications of the subunit cause a reduction in expression levels. When these are present at 100% of the polymer the yields can be seriously reduced as seen with the Caf1^His^ mutant used here. When expressed as a minor component in a mosaic the yields return to acceptable levels. Since the Caf1 subunit is only 6 nm long a 10 μm diameter cell will interact with hundreds or thousands of Caf1 subunits so even if they are present at 1% of a mosaic polymer, poorly expressing versions are likely to be biologically active. Finally, some motifs exhibit synergy when in close proximity to each other, for example the RGDS and PHSRN motifs of fibronectin [38, 39]. Due to the 6 nm subunit repeat, the production of mosaic polymers will result in the random close proximity of Caf1 subunits harbouring different motifs in a way that a mixture of single subunit polymers might not. Therefore, the ability to produce, mosaic polymers with defined content increases the utility of Caf1 as a biomaterial.

### Mosaic polymers can trigger complex bioactivity

The formation of bone by primary hBMSCs was used as a case study for testing the bioactivity of the mosaic polymers. Normally, differentiation of hBMSCs into osteoblasts and subsequent bone formation requires the presence of a cocktail of supplements in the serum [40]. However, it has been shown previously that two peptide sequences from OPN and BMP2 could promote bone nodule formation in routine cell culture conditions [30]. These peptides were engineered into the Caf1 scaffold and combined through co-expression as a Caf1 mosaic polymer. hBMSCs were seen to adhere to the mosaic polymer, and over time the differentiation of these cells into osteoblasts and deposition of minerals onto the substrate was observed. Therefore, the mosaic polymers are bioactive and trigger the desired cell activity. Moreover, as the mosaic polymers facilitate both adhesion and differentiation without the use of extra components in the serum, their use is simpler than traditional methods. Caf1 has the capacity to incorporate many different bioactive peptide motifs, and by combining subunits containing these motifs together as mosaic polymers, complex functionalised biomaterials can be produced that could be tailored for many cell types and applications.

### Mosaic Caf1 polymers form from a pool of periplasmic Caf1M:Caf1 complexes

**Fig. 7:**
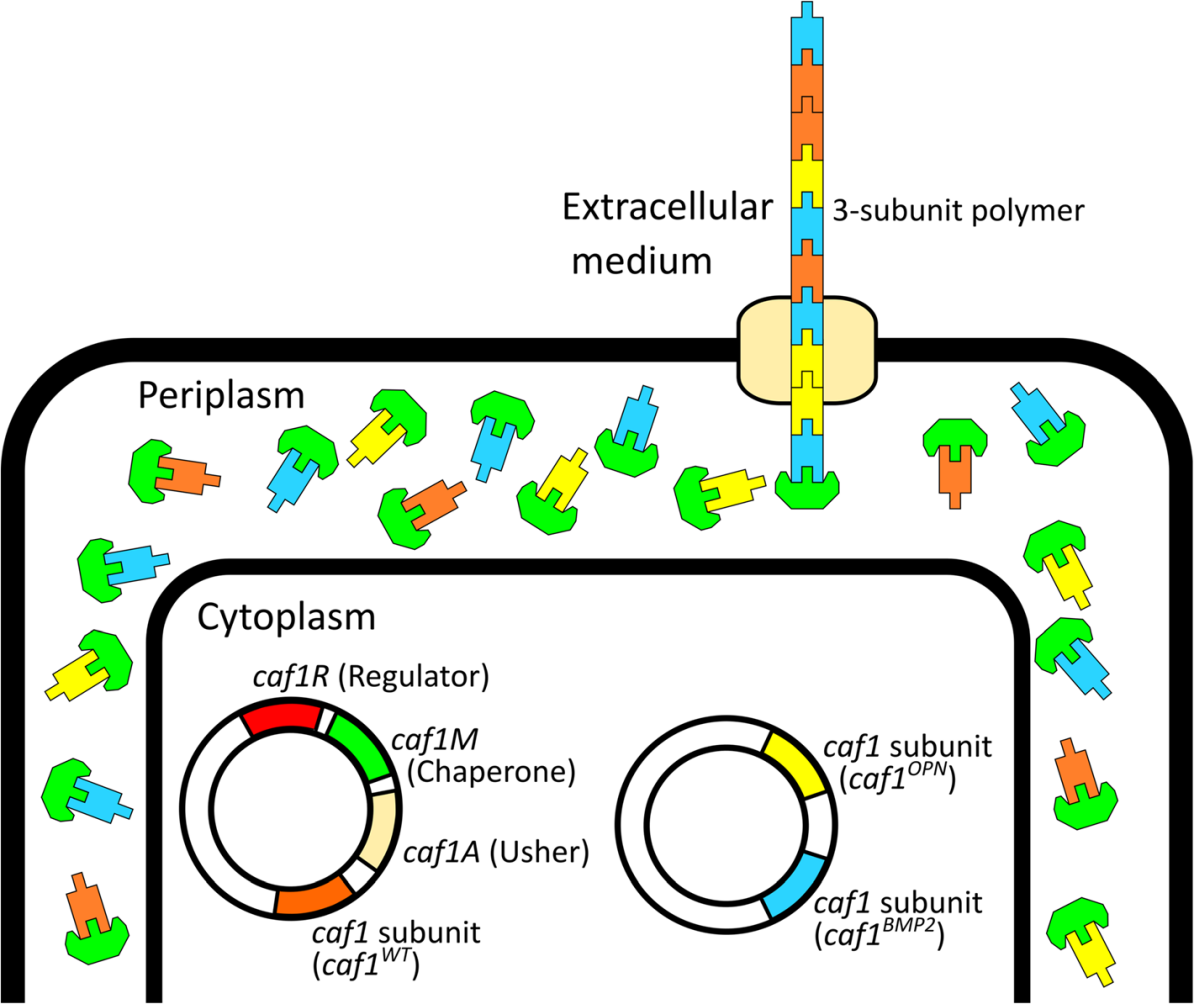
Model of mosaic Caf1 polymer production by bacterial cells. A diagram of a bacterium is shown with the cytoplasm, periplasm and extracellular medium sections labelled. The cytoplasm contains the pCOP and pBad2x-Caf1^OPN:BMP2^ plasmids needed for Caf1 mosaic polymer production where genes are colour coded as follows: *caf1R* regulator (red), *caf1M* chaperone (green), *caf1A* usher (tan), *caf1*^*WT*^ subunit (orange), *caf1*^*OPN*^ mutant subunit (yellow) and *caf1*^*BMP2*^ mutant (cyan). Caf1M and Caf1 subunits are targeted to the periplasm and form chaperone:subunit complexes. These complexes are assembled into a Caf1 polymer by the Caf1A usher, which resides in the outer membrane. The usher does not discriminate between the different types of Caf1 subunit, and so the polymer formed contains a random mixture of the expressed subunits, and is thus a three-subunit mosaic heteropolymer

The results of this study suggest that, upon co-expression of the two *caf1* subunit genes, there exists a pool of Caf1M chaperone bound Caf1 subunits in the periplasm which is assembled, presumably at random, by the Caf1A usher into polymers which are secreted outside of the bacterium (Fig. 7). Furthermore, when we constructed a pBad plasmid harbouring two *caf1* genes, induction of gene expression from this plasmid resulted in the simultaneous production of three Caf1 subunits when co-transformed into *E. coli* alongside a plasmid harbouring the natural but not overexpressing Caf1 operon. The results of this study have shown that co-expression of two subunits leads to true mosaic heteropolymers, and so it is assumed that the co-expression of three subunits leads to a 3-subunit mosaic heteropolymer. Our results would indicate that, depending on the limit of protein expression that can be tolerated by the bacterium, multiple Caf1 subunits could be co-expressed to form the Caf1M:Caf1 pool, and hence be assembled by Caf1A into complex mosaic polymers (Fig. 7). It is reasonable to assume that this system could be further expanded to add extra Caf1 subunits that could further increase complexity and functionality to the Caf1 polymers.

### Benefits of the Caf1 biomaterial

The controlled combination of multiple bioactive signals or functional sites (e.g. protease sites or reactive groups such as cysteine or biotin) is difficult to achieve in currently available polymeric materials but simple to achieve in Caf1. Whilst natural decellularised ECM has a high bioactivity, it is difficult to fully characterise the material and it can vary from batch to batch [41–43]. Synthetic and natural materials are often functionalised through covalent attachment of peptides [36, 42–45], which can be expensive and challenging if multiple activities are required. In contrast, highly functionalised Caf1 polymers can be produced through bacterial fermentation of *E. coli* transformed with the plasmids described here. This avoids the problems associated with the synthesis, purification and attachment of multiple peptides to a material, and instead exploits the advantages of using a biological “cell-factory” as a production mechanism, placing Caf1 as a clear example of an engineered living material. Moreover, unlike the Curli protein ELM [1], Caf1 adopts an Ig-like fold [10] rather than forming amyloid fibres, allowing its use in a wider range of biomedical applications, in addition to nanotechnological applications. Further improvements to the system should focus on removing the need for arabinose as an inducer, in order to further reduce the costs of Caf1 bioproduction. Additionally, the application of further synthetic biology techniques, such as genetic circuits, may allow even greater control over the biogenesis and complexity of the Caf1 material, as has been shown previously with other ELMs [1]. In light of these advantages, engineered Caf1 polymers would appear to be a promising new addition to the array of biomaterials currently available to those interested in 3D tissue culture and tissue engineering, as well as in the field of biofilm engineering.

## Conclusions

In this work, we have used gel electrophoresis and transmission electron microscopy to demonstrate that co-expression of *caf1* subunits leads to the formation of mosaic heteropolymers. The benefits of these polymers is twofold: multiple bioactive motifs can be combined to make a multifunctional biomaterial, and bioactive subunits can be diluted to optimal levels. To demonstrate the utility of these polymers, osteopontin and bone morphogenetic protein 2 motifs were incorporated into a single Caf1 mosaic polymer, and their activity triggered the early stages of bone formation by primary human bone marrow stromal cells. A synthetic biology approach then allowed the construction of a 3-subunit mosaic polymer, demonstrating how the system could be expanded to introduce further functionalities into the Caf1 polymers. These results show how the production of engineered Caf1 mosaic polymers provides a simple route towards the creation of highly functionalised, designed biomaterials for use in various biomedical applications, such as 3D tissue culture and wound healing.

## Methods

### Plasmids and cloning

In this study, five new Caf1 mutants were produced: (1) *caf1*^*A5I*^, where alanine 5 in the linking β-strand is mutated to isoleucine; (2) *caf1*^*His*^, where a hexa-His tag is attached through a 10 amino acid flexible linker to the N-terminus of Caf1 (18-amino acids total); (3) *caf1*^*Cys*^, where a single cysteine residue was inserted at the N-terminus of Caf1; (4) *caf1*^*OPN*^, where a 7 amino acid sequence corresponding to the α5β1 integrin recognition site of osteopontin [23] was added to the N-terminus of Caf1 via a 6 amino acid flexible linker (13 amino acids total); and (5) *caf1*^*BMP2*^, where a 20 amino acid peptide motif corresponding to the knuckle epitope of bone morphogenetic protein 2 [29, 46] was inserted at the N-terminus of Caf1. The insertion site of mutations (2)–(4) was after the signal peptide, immediately before the start of the N-terminal linking β-strand. The protein sequences of mutants are shown in Additional file 1: Table S1. With the exception of *caf1*^*A5I*^, all mutant *caf1* genes were synthesised by GeneArt (Thermo Fisher Scientific) in the pBad33SD vector described previously [15].

For expression of Caf1 homopolymers, a plasmid (pT7-Caf1 Operon, pT7-COP, previously described as pGEM-T Caf1 [15]) containing all the genes of the Caf1 operon (*caf1R, caf1M, caf1A* and *caf1*), preceded by a T7 promoter, was used. We have observed that, when combined with a T7 promoter, deletion of *caf1R* (the *caf1* regulatory protein [47]) increases *caf1* expression. Therefore, the pT7-COP plasmid was further modified by *caf1R* deletion*,* to produce pT7-COPΔR. pCOP was produced by substitution of the T7 promoter with a random 20 nucleotide sequence. Deletion of the T7 promoter from the pT7-COP plasmid lowers *caf1* expression, so that it is only driven from the native promoters within the operon, with no overexpression.

For construction of the *caf1*^*A5I*^ mutant (pT7-COPΔR-Caf1^A5I^), the pT7-COPΔR plasmid was used as a template for PCR, with primers listed in Additional file 1: Table S2. To construct the pBad2x plasmid, the pBad33SD plasmid was used as a template and linearised by PCR. Linear inserts corresponding to the c*af1*^*OPN*^ and *caf1*^*BMP2*^ mutant *caf1* genes were generated by PCR. All species were purified by gel extraction from 0.8% w/v agarose gels following electrophoresis using a Monarch gel extraction kit (NEB) according to the manufacturer’s protocol. Linearised species were re-ligated using the sequence and ligation independent cloning (SLIC) method [48]. *caf1*^*His*^*, caf1*^*OPN*^ and *caf1*^*BMP2*^ mutant *caf1* genes were transferred from the pBad33SD vector to the pT7-COP plasmid using the InFusion HD cloning kit (Takara Clontech). Primer sequences are shown in Additional file 1: Table S2.

### Protein expression and purification

BL21(DE3) *E. coli* cells (NEB) were transformed with pT7-COP or pT7-COPΔR-Caf1^A5I^, either on their own or as a co-transformation with pBad-Caf1^His^. Expression cultures were then made by using single colonies of these transformants to inoculate Terrific Broth media. To induce expression of the subunit on the pBad plasmid, L-arabinose was added to the culture to a final concentration of 0.9% w/v. Wild-type Caf1 protein (Caf1^WT^) was produced using a pT7-COP/pBad-Caf1^His^ co-transformed culture that was not induced with arabinose. Cultures were grown at 35 °C for 22 h to produce Caf1 polymers. Following expression, cultures were centrifuged at 11325 x g for 15 min to remove the cell pellet. Pellets were discarded, leaving the diffuse “flocculent layer” which results from *caf1* expression [49] and the supernatant. This material was stirred at room temperature overnight to extract Caf1 polymers from the flocculent layer. The solution was then centrifuged again at 48384 x g for 50 min to pellet remaining flocculent and insoluble material. The supernatant containing Caf1 was concentrated to ~ 10 mL using two Minimate TFF 500 kDa molecular weight cut off (MWCO) capsules (Pall) arranged in tandem, then washed 1–4 times by diafiltration with 200 mL phosphate buffered saline (PBS). For polymers containing a His-tagged subunit, the retentate was then applied to a 5 mL HisTrap column (GE Healthcare) pre-equilibrated in 50 mM Tris-HCl pH 7.5, 150 mM NaCl, 20 mM imidazole and eluted from the column in the same buffer except that the imidazole concentration was 250 mM. Elution fractions containing the protein were pooled then concentrated and simultaneously buffer exchanged into phosphate buffered saline (Sigma Aldrich) using a 100 kDa MWCO Proteus X-spinner (Generon). Caf1^His:Cys^ and Caf1^OPN:BMP2^ mosaic polymers were purified as described previously [13, 15]. Bacterial endotoxin was removed from the Caf1^WT^ and Caf1^OPN:BMP2^ proteins used in cell assays by passing them through 48 mL Captocore 700 resin (GE Healthcare) packed into a Tricorn 10/600 column (GE Healthcare). This was confirmed to be < 10 endotoxin units/mL using a Pyrogene assay (Lonza). The final yields of each protein were as follows: Caf1^WT^, 207 mg/L; Caf1^A5I^, 137 mg/L; Caf1^WT:His^, 25.4 mg/L; Caf1^A5I:His^, 2.26 mg/L; Caf1^His:Cys^, 17.27 mg/L; Caf1^OPN:BMP2,^ 26.8 mg/L.

### Cysteine labelling

Caf1^His:Cys^ protein was buffer exchanged from PBS into a solution containing 50 mM Tris pH 7, 150 mM NaCl and 5 mM TCEP using a PD-10 column (GE Healthcare). Elution fractions containing the protein were then concentrated using Vivaspin 20,100 kDa MWCO centrifugal concentrators, and then further concentrated to approximately 4 mg/mL using Proteus X-spinner 100 kDa MWCO concentrators (Generon). EZ-link Maleimide-PEG-Biotin (ThermoFisher Scientific) was added in a 20-fold molar ratio and the solution incubated overnight at room temperature. Following incubation, to remove unreacted label, the protein was applied to a second PD-10 column and eluted in 50 mM Tris pH 7, 150 mM NaCl and 5 mM TCEP.

### Thermal melts

Thermal melts were measured in triplicate by circular dichroism spectropolarimetry (CD). 1 mg/mL Caf1 protein in PBS was added to a 1 cm path length quartz-Suprasil cuvette (Hellma 105–201-QS), and the near UV-CD signal measured at 290 nm (corresponding to a peak in the Caf1 near UV-CD spectrum [15]) while the temperature was increased at 2 °C/min between 20 and 95 °C. The CD signal was converted into the fraction of protein folded, using the signal at 20 °C to represent fully folded protein and the minimum CD signal to represent fully unfolded protein. To determine the melting temperature (T_m_) of the protein, the CD signal was converted into a first order derivative plot and the peak minimum (equivalent to the mid-point transition where 50% of the protein is unfolded) recorded as the T_m_.

### Electron microscopy

Solutions were prepared as follows: proteins were diluted to a concentration of 10 μg/mL in water, 10 nm Ni-NTA-Nanogold (Nanoprobes) and 20 nm gold – streptavidin conjugate (Expedeon) as supplied were diluted fivefold. Nickel electron microscope grids with thin film carbon supports were glow discharged and placed carbon side down in 20 μL of the protein solution for 1 min, then placed in the 20 nm gold – streptavidin solution for 5 min. After this incubation, the grids were washed three times in water for 10 s each, then added to the Ni-NTA-Nanogold solution for 5 min, washed a further three times for 10 s each, then placed into a solution of 2% uranyl acetate for 2 min. After each incubation, grids were dried with filter paper. Grids were then visualised using a Philips CM100 transmission electron microscope (EM Research Services, Newcastle University) operated at 100 kV. Images were taken at a magnification of 92,000x and recorded in tagged image file format (TIFF).

### Cell biology

Human mesenchymal stem cells from individual donors were supplied by Lonza having been previously characterised as CD105, CD166, CD29, CD44 positive and negative for CD14, CD34 and CD45. Cells were routinely cultured in alpha Modified Eagle Medium (αMEM, Thermo Fisher Scientific) supplemented with 10% v/v FCS (First Link (UK)), 100 units/ml penicillin, 100 μg/mL streptomycin (both Thermo Fisher Scientific) and 1 ng/mL FGF-2 (PeproTech). Cells were grown at 37 °C in a humidified incubator with an atmosphere of 5% CO2 and passaged at approximately 70% confluence with all experiments performed before passage six. To assess the influence of the Caf1 polymer, 24-well plates were coated as follows: proteins at ~ 4 mg/mL were diluted in PBS (Sigma D8537) to 100 μg/ml and sterile filtered at 0.2 μm. 200 μl was added to wells (Greiner 24 well glass bottomed plate, product code 662892) and incubated overnight at room temperature. These plates were used since we have noticed that Caf1 adheres better to glass than plastic [15]. Solution was aspirated and wells washed with 2 × 500 μl PBS, then air dried for 20 mins and stored at 4 °C. 50,000 cells were plated into each well and cultured for up to 14 days with the culture medium described above refreshed every 3 or 4 days. To study gene expression, total RNA was isolated using the Direct-zol RNA Kit (Zymo Research) as directed by the manufacturer and 450 ng of each sample used as the template for cDNA synthesis in the QuantiTect Reverse Transcription Kit (Qiagen). Quantitative PCR was performed using the Sybr Green approach with the 2X QuantiFast SYBR Green PCR Kit (QIAGEN) and relative quantitation achieved using the comparative Ct (2-ΔΔ CT) method. The primers used in this analysis were HPRT1 (forward – TGACACTGGCAAAACAATGCA, reverse - GGTCCTTTTCACCAGCAAGCT), Runx2 (forward – GGTTAATCTCCGCAGGTCAC; reverse – GTCACTGTGCTGAAGAGGCT) and BMP2 (Qiagen Quantitect® Primer Assay).

## Additional file

Additional file 1: Supplementary tables and figures. **Tables S1-S2.** and **Figures S1-S3.** (PDF 706 kb)

## Data Availability

The datasets used and/or analysed during the current study are available from the corresponding author on reasonable request.

## Abbreviations

BMP2: Bone Morphogenetic Protein 2
ELM: Engineered Living Material
hBMSCs: Human Bone Marrow Stromal Cells
OPN: Osteopontin
SMAD: Sma and MAD related protein
TEM: Transmission Electron Microscopy
WT: Wild-type
