# Supplementary material for: Engineered mosaic protein polymers; a simple route to multifunctional biomaterials
Source: J Biol Eng. 2019 Jun 18;13:54. doi: 10.1186/s13036-019-0183-2 (PMC6582577; doi:10.1186/s13036-019-0183-2)
Supplement: Supplementary file 1 — Supplementary tables and figures. Tables S1-S2. and Figures S1-S3. (PDF 706 kb) [file 13036_2019_183_MOESM1_ESM.pdf]

Supplementary Information

**Mutant Name**	**Sequence**	**Subunit Molecular Weight (kDa)**
**Caf1^{WT}**	ADLTASTTATATLVEP	**15.6**
**Caf1^{A5I}**	ADLT*I*STTATATLVEP	**15.6**
**Caf1^{His}**	A*SSHHHHHHGGGGSGGGGS*DLTASTTATATLVEP	**17.2**
**Caf1^{Cys}**	A*C*DLTASTTATATLVEP	**15.7**
**Caf1^{OPN}**	AD*SVVYGLRGSGSGS*LTASTTATATLVEP	**16.8**
**Caf1^{BMP2}**	AD*KIPKASSVPTELSAISTLYL*LTASTTATATLVEP	**17.6**

Table S1: Sequences and sizes of Caf1 mutant subunits used in this study. Mutations are written in italics.

**Primer**	**Sequence**
A5I Forward	agcaccactgcaacggcaac
A5I Reverse	cgttgcagtggtgctaatagttaaatctgccgcattagcag
pBad2x Vector Forward	tctagagtcgacctgcagg
pBad2x Vector Reverse	acagtagagagttgcgataaaaag
pBad2x Insert 1 Forward	gcaactctctactgtttctccatacccgtttttttggg
pBad2x Insert 1 Reverse	aacgggtatggagaattattactgattgctaacggtcac
pBad2x Insert 2 Forward	ttctccatacccgtttttttggg
pBad2x Insert 2 Reverse	caggtcgactctagattattactgattgctaacggtcac
pT7-COP Vector Forward	gaattccccaatcactagaattcg

pT7-COP Vector Reverse	atattacctctatcgaataatcc
pT7-COP Insert Forward	cgatagaggtaatatatgaaaaaaatcagc
pT7-COP Insert Reverse	gtgattggggaattcttactgattgctaac
pT7-COP T7 Substitution Forward	tctagccgttcgaattgggcccgacgtc
pT7-COP T7 Substitution Forward	agaacaagaacaattcactggccgtcgttttac
pT7-COP Caf1R Deletion Forward	taatcctaatgttacagaatataacccaaatcaaaataatag
pT7-COP Caf1R Deletion Reverse	gtaacattaggattaccaaagag

Table S2: Primer sequences used in this study.

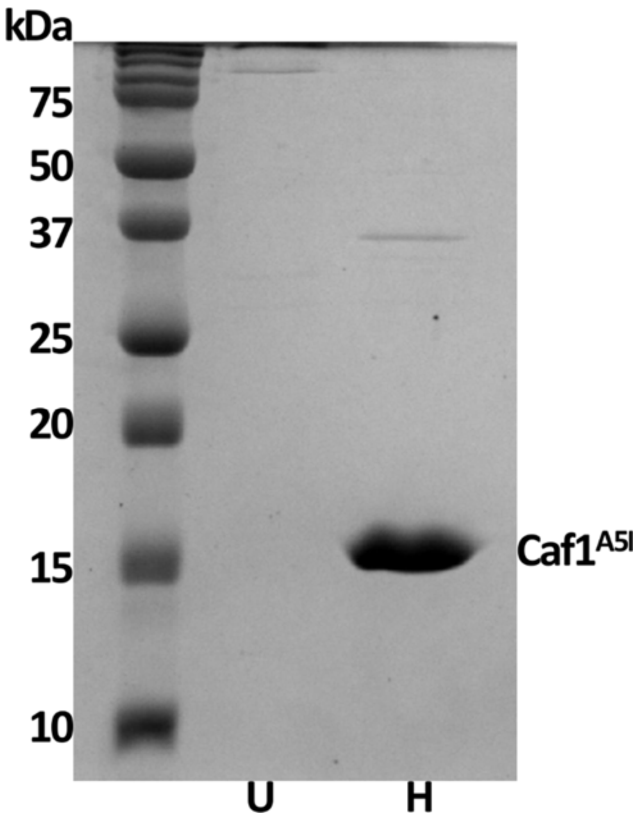

Figure S1: SDS-PAGE analysis of the Caf1 A5I mutant. Samples of the purified protein were added to an SDS containing sample buffer. One sample (H) was heated to 100°C for 5 minutes before gel loading while the other sample was kept at room temperature (U). The position of the Caf1 A5I monomeric subunit is highlighted at 15.6 kDa and can be seen in the H sample.

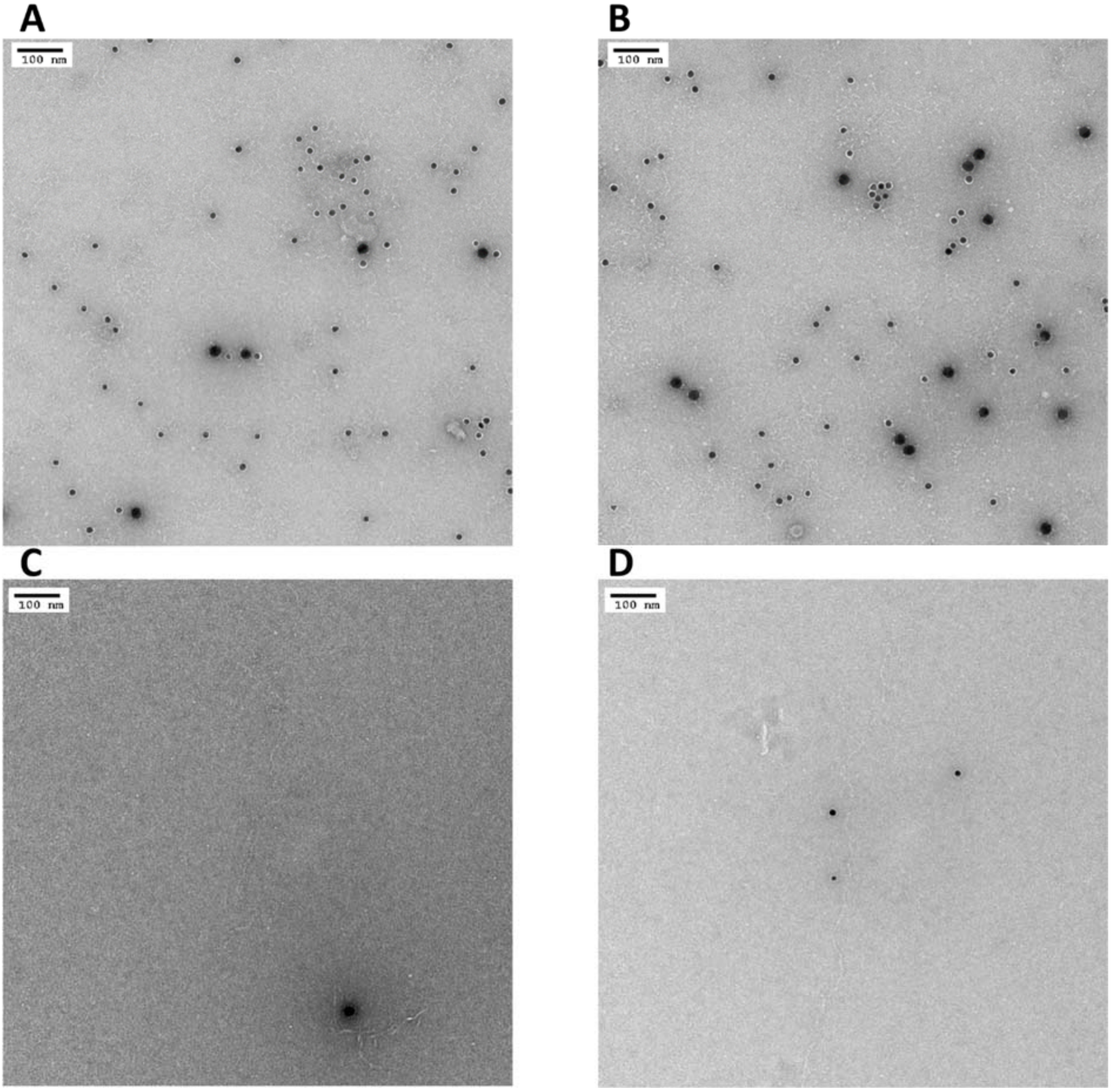

Figure S2: Transmission electron microscopy images of Caf1 mosaic polymers labelled with gold nanoparticles. Negative stain electron micrographs of (**A** and **B**) the His-tagged, biotinylated cysteine containing mosaic polymer ($Caf1^{His:Cys(Biotin)}$) and (**C** and **D**) the $Caf1^{WT}$ polymer. Polymers were probed with Nickel-NTA-10 nm gold particles and Streptavidin-20 nm gold particles. Images were taken at 92000x magnification.

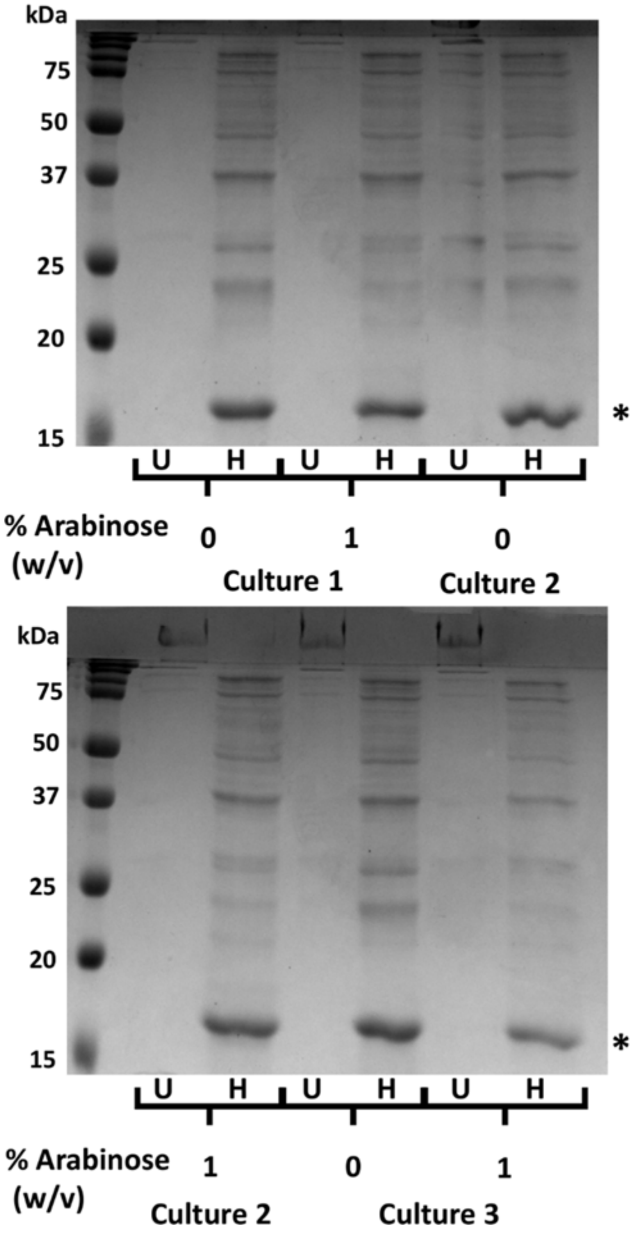

Figure S3: Expression tests of pT7-COP/pBad2x $^{OPN:BMP2}$ in *E. coli* BL21(DE3) cells. Cultures of *E. coli* BL21(DE3) cells transformed with pT7-COP and pBad2x-Caf1$^{OPN:BMP2}$ were grown in triplicate for 22 h in the presence and absence of 1% w/v arabinose. Samples of the extracellular fraction (flocculent layer and supernatant) were then either heated to 100°C (H) or incubated at room temperature (U) for 5 minutes in SDS containing buffer and applied to the gel. Caf1 monomer bands are highlighted with a star.
